# PReS-FINAL-2059: Th17 cells highly enriched in peripheral blood in children with HLA B27-associated arthritis and systemic onset juvenile idiopathic arthritis

**DOI:** 10.1186/1546-0096-11-S2-P71

**Published:** 2013-12-05

**Authors:** I Turtsevich

**Affiliations:** 1 Saint-Petersburg State Pediatric Medical University, Saint-Petersburg, Russian Federation

## Introduction

According to modern concepts of JIA as seen as generalizing term that brings together a heterogeneous group of chronic diseases of joints with different etiology, pathogenesis and immunogenetic origin, different nosology and controversial prognosis.

Population of Th17 cells involved in pathogenesis of many autoimmune and chronic diseases due to the ability to destroy extracellular matrix.

## Objectives

To optimize the diagnosis and treatment of different types of JIA by identifying influence of the main immunological factors of Th17 cells differentiation of lymphocytes.

## Methods

PB samples were obtained from 100 patients with different subtypes of JIA and 20 PB samples from healthy control. The bases of quantitative evaluation of Th17 cells were taken directly determination of CCR6 and CCR4 in PBMC by flow cytometry. Cytokines, such as IL-1β, IL-6, IL-17A and tnfα were determined in serum samples of the patients by ELISA.

## Results

Highest level of IL-17+T cells in peripheral blood was determined in children with active HLA B27 associated arthritis (p = 0,002) and systemic onset JIA (p = 0,005). As active oligoarticular and polyarticular subtypes of JIA, it was observed less high level of these cells, if compare with HLA B27+ and systemic onset arthritis. Level of IL-17A IL-1β, IL-6 (in contrast with tnfα level), was increased in patients with active HLA B27+ arthritis, if compare with other subtypes of JIA (p < 0,05). In patients with complete clinical and laboratory remission level of IL-17+T cells was reduced and almost did not differ from the control group. We also found statistically significant correlation between IL-6 level, RO+CCR6+ and the presence of osteoporosis in children with JIA. During therapy with tocilizumab expression of CCR6 and CCR4 on PBMC was decreased in patients with systemic onset JIA.

## Conclusion

Our data suggest that Th17 cells and their cytokines play a crucial role in pathogenesis of JIA, especially in systemic onset and HLA B27 associated arthritis. Treatment with anti-IL-6 agents (tocilizumab) effectively suppress Th17 differentiation pathway of lymphocytes.

## Disclosure of interest

None declared.

